# Reliability and validity of a novel attention assessment scale (broken ring enVision search test) in the Chinese population

**DOI:** 10.3389/fpsyg.2024.1375326

**Published:** 2024-05-09

**Authors:** Yue Shi, Yi Zhang

**Affiliations:** Department of Rehabilitation Medicine, Third Affiliated Hospital of Soochow University, Changzhou, China

**Keywords:** attention, attention assessment, broken ring enVision search test, reliability, validity, age, education level, gender

## Abstract

**Background:**

The correct assessment of attentional function is the key to cognitive research. A new attention assessment scale, the Broken Ring enVision Search Test (BReViS), has not been validated in China. The purpose of this study was to assess the reliability and validity of the BReViS in the Chinese population.

**Methods:**

From July to October 2023, 100 healthy residents of Changzhou were selected and subjected to the BReViS, Digital Cancelation Test (D-CAT), Symbol Digit Modalities Test (SDMT), and Digit Span Test (DST). Thirty individuals were randomly chosen to undergo the BReViS twice for test–retest reliability assessment. Correlation analysis was conducted between age, education level, gender, and various BReViS sub-tests including Selective Attention (SA), Orientation of Attention (OA), Focal Attention (FA), and Total Errors (Err). Intergroup comparisons and multiple linear regression analyses were performed. Additionally, correlation analyses between the BReViS sub-tests and with other attention tests were also analyzed.

**Results:**

The correlation coefficients of the BReViS sub-tests (except for FA) between the two tests were greater than 0.600 (*p* < 0.001), indicating good test–retest reliability. The Cronbach’s alpha coefficient was 0.874, suggesting high internal consistency reliability. SA showed a significant negative correlation with the net score of D-CAT (*r* = −0.405, *p* < 0.001), and a significant positive correlation with the error rate of D-CAT (*r* = 0.401, *p* < 0.001), demonstrating good criterion-related validity. The correlation analysis among the results of each sub-test showed that the correlation coefficient between SA and Err was 0.532 (*p* < 0.001), and between OA and Err was-0.229 (*p* < 0.05), whereas there was no significant correlation between SA, OA, and FA, which indicated that the scale had good informational content validity and structural validity. Both SA and Err were significantly correlated with age and years of education, while gender was significantly correlated with OA and Err. Multiple linear regression suggested that Err was mainly affected by age and gender. There were significant differences in the above indexes among different age, education level and gender groups. Correlation analysis with other attention tests revealed that SA negatively correlated with DST forward and backward scores and SDMT scores. Err positively correlated with D-CAT net scores and negatively with D-CAT error rate, DST forward and backward scores, and SDMT scores. OA and FA showed no significant correlation with other attention tests.

**Conclusion:**

The BReViS test, demonstrating good reliability and validity, assessing not only selective attention but also gauging capacities in immediate memory, information processing speed, visual scanning, and hand-eye coordination. The results are susceptible to demographic variables such as age, gender, and education level.

## 1 Introduction

Attention is the foundation of all cognitive functions, the prerequisite for continuous information processing, and a gateway for the flow of information to enter the brain and undergo selection (Petersen and Posner, 2012). Precise and accurate assessment of attentional functions is key in cognitive research and a precondition for the rehabilitation of cognitive disorders. In clinical neuropsychology, visual search tasks (VSTs) are frequently used to evaluate selective visual attention deficits in patients with neurological conditions (Eglin et al., 1989; Luck et al., 1989; Utz et al., 2013). These typically include paper-and-pencil target cancellation tasks such as the Attention Matrix (Della Sala et al., 1992), Ruff 2&7 Selective Attention Test (Marioni et al., 2012), Letter Cancellation Test (Uttl and Pilkenton-Taylor, 2001), and the Visual Spatial Attention subtest in the Oxford Cognitive Screen (Demeyere et al., 2015), which are effective tools for detecting attention deficits post-stroke. However, existing VSTs do not take into account the potential impact of stimulus layout and crowding on the test results of participants. Facchin et al. developed a novel attention assessment scale—the Broken Ring enVision Search Test (BReViS) to evaluate attentional functions (Facchin et al., 2023). It assesses different components of attention including selective attention, the visual–spatial orientation of attention, and focal attention involving crowding phenomena, and is a novel open-ended paper-and-pencil assessment tool.

While studies have shown the effectiveness and applicability of the BReViS test in the Italian population and provided specific Italian normative data, its suitability for the Mainland Chinese population is yet to be concluded. Therefore, this study aims to examine the reliability and validity of the BReViS test in the healthy Chinese population and to analyze the characteristics of its preliminary application, in the hope of finding a simple and feasible tool for the clinical environment to assess neuropsychological patients’ attention deficits and provide a basis for the assessment and rehabilitation treatment of attentional disorders.

## 2 Sample and methods

### 2.1 Study procedure

General Information: From July to October 2023, a total of 100 healthy residents, including staff and accompanying personnel from the First People’s Hospital of Changzhou and residents of Tianning and Xinbei districts of Changzhou, were selected. The cohort comprised 47 males and 53 females; ages ranged from 19 to 84 years, with an average age of (52.35 ± 22.01) years; years of education ranged from 2 to 20 years, with an average of (12.39 ± 3.86) years. Of these, the number of people with 2 years of education was 1.

Inclusion criteria: Age 19–84 years; Right-handed; Normal or corrected-to-normal vision.

Exclusion criteria: Auditory, visual, or speech impairments; Past history of neurological or psychiatric diseases (including brain injury, stroke, clinically diagnosed dementia, depression, etc.); History of addiction to tobacco, alcohol, or addictive drugs.

Grouping method: In order to make between-group comparisons between different ages, education levels and genders, the subjects were divided into 4 groups according to different ages in the statistical analyses, with those aged 18–34 years classified as the youth group, those aged 35–49 years classified as the young-adult group, those aged 50–65 years classified as the middle-aged group, and those older than 65 years classified as the senior group. Similarly, they were divided into four groups according to their education level: those with 1–6 years of education were classified as the elementary group, those with 7–9 years of education were classified as the middle school group, those with 10–12 years of education were classified as the high school/vocational group, and those with more than 12 years of education were classified as the college/university and above group. They were divided into male and female groups by gender. Demographic characteristics of the groups are reported in Table 1. Thirty subjects were randomly selected as the retesting group and the BReViS test was administered again after 2 weeks. There were 30 subjects in the retesting group, of whom 14 were male and 16 were female; their ages ranged from 19 to 72 years, with a mean of (44.07 ± 15.67) years; and their years of education ranged from 6 to 19 years, with a mean of (13.86 ± 2.81) years.

**Table 1:** Demographic characteristics of the patients’ sample.

Age	19–34		35–49		50–65		>65		Tot.
School	F	M	F	M	F	M	F	M	
1–6	0	0	0	0	1	0	5	4	10
7–9	0	0	0	1	2	2	7	6	18
10–12	0	1	0	1	1	2	11	8	24
>12	20	15	4	3	1	0	1	4	48
Tot.	20	16	4	5	5	4	24	22	100

F = female; M = male.

### 2.2 Measurements and applied questionnaires

#### 2.2.1 The BReViS test

It was developed by Facchin et al. (2023). We have obtained authorization from the original authors to use it. The test consisted of four cancellation quiz cards, each consisting of five rows of circles with notches in different orientations arranged in different layouts and degrees of crowding, with 25 targets per card and randomly defined target locations. Subjects were asked to identify and cross out all the targets on each card that had the same notch orientation as the circles shown at the top of the card, and to record the execution time, number of omissions, self-corrections, and errors crossings for the completion of the 4 test cards. The performance time for each quiz card was calculated based on the execution time and omissions for each card. The calculation formula is as follows:


$$\mathrm{Performance}\;\mathrm{time}=\frac{25\times \mathrm{Execution}\;\mathrm{time}}{25-\mathrm{omissions}}$$

By combining the execution times of the four test cards, the following four indices are calculated: Selective Attention (SA), Orientation of Attention (OA), Focal Attention (FA), and Total Errors (Err).

SA represents the capacity to suppress irrelevant stimuli (distractors) and solely select relevant stimuli (targets) under the simplest conditions. It directly corresponds to the performance time of the first card (linear layout, low crowding), which is less affected by random arrays and crowded displays. SA = Performance time for the first card. Higher SA index values suggest lower efficiency of selective attention.

OA refers to the strategic direction of visual attention, which is the capacity to guide selective visual attention with effective endogenous strategies throughout the visual scene (Connor et al., 2004), one of the two components of visual–spatial attention measured by BReViS. High OA index values indicate an inability to follow effective endogenous strategies during the visual search process, necessitating exogenous cues to perform the task correctly. It is calculated with the following formula using the performance time of each card:


$$OA=\frac{\mathrm{Card}3+\mathrm{Card}4}{2}-\frac{\mathrm{Card}1+\mathrm{Card}2}{2}$$

FA can be interpreted as the ability to adjust the focus of attention based on the position of stimuli within the array, another component of visual–spatial attention (Castiello and Umilta, 1990). It corresponds to the comparison between two levels of crowding: high and low. High FA index values suggest a higher sensitivity to crowding. It is calculated with the following formula using the performance time of each card:


$$FA=\frac{\mathrm{Card}2+\mathrm{Card}4}{2}-\frac{\mathrm{Card}1+\mathrm{Card}3}{2}$$

The Err index represents the overall errors made across all sub-tests. Err = Total number of errors across all four test cards.

#### 2.2.2 Other attention tests

The Digit Cancellation Test (D-CAT) is used to measure selective attention (Hatta et al., 2004). Participants were required to locate and strike through the number preceding the number 3 from a random sequence of numbers 1–9, with the time taken to complete the test recorded. Net scores and error rates are calculated based on the number of correct cancelations, omissions, and mistakes. Higher net scores and lower error rates indicate better selective attention.

The Symbol Digit Modalities Test (SDMT) was published by Aaron Smith in 1973 and revised in 1982 to assess speed of information processing, visual scanning ability, and hand-eye coordination (Strober et al., 2019). This test involves an encoding key of 9 different abstract symbols, each associated with a number. Participants must write the number corresponding to each symbol as quickly as possible within 90 s. Scoring is based on the number of correct symbols and reversed symbols. Higher scores indicate better speed of information processing, visual scanning ability, and hand-eye coordination.

The Digit Span Test (DST) is a commonly used psychological assessment tool that measures short-term memory and attention span (Park and Lee, 2019). In its traditional form, the Digit Span Test consists of two parts: forward digit span and backward digit span. This test evaluates the participant’s ability to recall a sequence of numbers in the correct order both forwards and backwards after the tester reads them out. Participants repeat a series of random numbers at a rate of one number per second, starting with a sequence of 3 numbers and increasing in length up to 12 numbers or until two consecutive errors are made. One point is scored for each correctly recalled sequence. The higher the scores on forward and backward digit span, the greater the capacity of immediate memory.

#### 2.2.3 Sample size calculation

This study mainly used correlation analysis and multiple linear regression analysis, so it was calculated using G*Power software 3.1 (Faul et al., 2009), correlation analysis input target effect size of 0.3, type I error of 5% (α = 0.05), and power of 80% (β = 0.20), and the sample size of 82 participants was calculated. Multiple linear regression analyses were conducted with an input independent variable of 3 (U = 3), effect size = 0.15 (F2 = 0.15), type I error of 5% (α = 0.05), and power of 80% (β = 0.20), resulting in a calculated sample size of 77 participants. The final sample size was 100 participants, taking into account an allowable 20% dropout rate.

#### 2.2.4 Experimental procedure

Participants filled out informed consent forms; They were subjected to the BReViS test and other attention tests. Among them, 30 were randomly selected to retake the BReViS test after two weeks. All tests were administered by the same physician.

### 2.3 Statistical analysis

SPSS 17.0 software was used for statistical analysis. Spearman’s correlation analysis was employed to assess the correlation between the BReVis test and other attention tests, as well as the correlation between each sub-test of the BReViS and age, educational level, and gender. Kruskal-Wallis test was used to compare the differences in the BReViS sub-test scores among different age and educational level groups, while Mann–Whitney U test was utilized to compare the differences between gender groups. Multiple linear regression analysis was conducted to investigate the influence of demographic characteristics on scale evaluation results, with statistical significance set at *p* < 0.05. Pearson correlation coefficient was employed to analyze the test–retest reliability of the BReViS; Cronbach’s α coefficient was used to indicate internal consistency, with a coefficient above 0.80 considered excellent, between 0.70 and 0.80 acceptable, and below 0.7 indicating poor reliability. The Kaiser-Meyer-Olkin (KMO) measure of sampling adequacy and Bartlett’s test of sphericity were employed to analyze the appropriateness of factor analysis, to validate the structural validity of the BReViS. Finally, correlation analyses between the results of the BReViS subtests were conducted using Spearman’s correlation analysis to test the content and structural validity of the scale.

## 3 Results

### 3.1 Descriptive results

The descriptive mean results on the four BReViS sub-tests scores are reported in Tables 2–4.

**Table 2:** Mean performance time (and SD) for each sub-test, divided by age group.

Sub-test	19–34	35–49	50–65	>65
SA	49.95 (12.36)	64.28 (14.37)	79.58 (18.56)	98.73 (27.77)
OA	30.06 (18.83)	30.44 (17.93)	26.50 (27.28)	28.89 (36.01)
FA	−0.69 (12.10)	−5.56 (10.14)	1.83 (13.72)	4.09 (17.52)
Err	9.33 (5.78)	13.56 (7.27)	19.89 (8.34)	22.41 (10.21)

**Table 3:** Mean performance time (and SD) for each sub-test, divided by education level group.

Sub-test	1–6	7–9	10–12	>12
SA	101.63 (22.02)	104.3 (26.86)	86.72 (26.76)	55.41 (18.90)
OA	39.05 (32.33)	17.28 (40.84)	36.88 (28.02)	27.85 (19.87)
FA	0.55 (18.44)	8.28 (17.33)	0.42 (17.16)	−0.73 (11.45)
Err	27.3 (11.37)	20.22 (12.07)	18.88 (7.96)	12.04 (7.82)

**Table 4:** Mean performance time (and SD) for each sub-test, divided by gender group.

Sub-Test	Male	Female
SA	76.66 (26.55)	76.07 (34.40)
OA	36.30 (26.78)	22.97 (28.42)
FA	−0.15 (14.71)	2.58 (15.23)
Err	14.06 (7.62)	19.00 (11.77)

### 3.2 Correlation analysis of age with the BReViS sub-tests

Age showed a positive correlation with both SA (*r* = 0.776, *p* < 0.001) and Err (*r* = 0.607, *p* < 0.001), with no significant correlation with the other sub-tests.

### 3.3 Comparison of different age groups

As shown in Table 5, analyses of multiple between-group comparisons across age groups showed significant differences in sub-test scores for SA and Err (*p* < 0.001). Detailed two-by-two intergroup comparisons highlighted significant differences in SA scores between the youth and middle-aged groups (adjusted *p* = 0.006), as well as between the youth and senior groups (adjusted *p* = 0.000). Similarly, Err scores differed significantly between the young and middle-aged groups (adjusted *p* = 0.005), and between the youth and senior groups (adjusted p = 0.000). Additionally, a distinct variance was observed in SA scores between the young-adult and senior groups (adjusted *p* = 0.017), as shown in Table 6.

**Table 5:** Analysis of variance between different age groups (Mean Rank).

Sub-test	Youth group	Young-adult group	Middle-aged group	Senior group	*p*
SA	22.53	41.11	58.22	72.72	0.000
OA	51.81	52.11	44.67	50.30	0.926
FA	47.72	36.22	53.44	54.89	0.301
Err	27.25	42.33	63.56	67.74	0.000

**Table 6:** Two-by-two comparison of SA and Err between different age groups.

Sample 1-Sample 2	SA			Err		
	Test Statistic	S.E	Adj.*p*	Test Statistic	S.E	Adj.*p*
1–2	−18.58	10.81	0.514	−15.08	10.80	0.976
1–3	−35.69	10.81	0.006	−36.31	10.80	0.005
1–4	−50.19	6.46	0.000	−40.49	6.45	0.000
2–3	−17.11	13.68	1.000	−21.22	13.67	0.723
2–4	−31.61	10.57	0.017	−25.41	10.57	0.097
3–4	−14.50	10.57	1.000	−4.18	10.57	1.000

1 = the youth group; 2 = the young-adult group; 3 = the middle-aged group; 4 = the senior group.

### 3.4 Correlation analysis of education level with the BReViS sub-tests

Years of education were negatively correlated with both SA (*r* = −0.715, *p* < 0.001) and Err (*r* = −0.502, *p* < 0.001), with no significant correlation with the remaining sub-tests.

### 3.5 Comparison of different education level groups

As shown in Table 7, analyses of multiple between-group comparisons across education level groups unveiled significant disparities in the scores for sub-tests SA and Err, while OA and FA did not exhibit such differences (*p* < 0.001). Detailed two-by-two intergroup comparisons highlighted significant differences in SA scores: the college/university and above group demonstrated significant disparities when compared with the elementary, middle school, and high school/vocational groups (adjusted *p* = 0.000 for all comparisons). Similarly, Err scores significantly differed between the college/university and above group and the elementary group (adjusted p = 0.000), as well as between the college/university and above group and both the middle school (adjusted *p* = 0.027) and high school/vocational groups (adjusted *p* = 0.006), as detailed in Table 8.

**Table 7:** Analysis of variance between different education level groups (mean rank).

Sub-test	Elementary group	Middle school group	High school/vocational group	College/University group and above	*p*
SA	77.05	76.97	62.73	28.93	0.000
OA	56.90	41.14	57.54	49.16	0.275
FA	49.10	61.17	50.33	46.88	0.361
Err	79.60	59.06	60.29	36.33	0.000

**Table 8:** Two-by-two comparison of SA and Err between different education level groups.

Sample 1-Sample 2	SA			Err		
	Test Statistic	S.E	Adj.*p*	Test Statistic	S.E	Adj.*p*
4–3	33.80	7.25	0.000	23.96	7.25	0.006
4–2	48.05	8.02	0.000	22.72	8.01	0.027
4–1	48.12	10.08	0.000	43.27	10.08	0.000
3–2	14.24	9.05	0.692	1.236	9.04	1.000
3–1	14.32	10.92	1.000	19.31	10.91	0.461
2–1	0.08	11.44	1.000	20.54	11.43	0.434

1 = the elementary group; 2 = the middle School group; 3 = the high school/vocational group; 4 = the college/university group and above.

### 3.6 Correlation analysis of gender with the BReViS sub-tests

Gender showed a negative correlation with OA (*r* = −0.251, *p* = 0.012) and a positive correlation with Err (*r* = 0.215, *p* = 0.032), with no significant correlation with SA and FA.

### 3.7 Comparison of the two gender groups

The comparison results between the two gender groups showed a significant difference in OA and Err (*p* < 0.05), while no significant difference was observed in SA and FA, as detailed in Table 9. Combining the results from Table 4, it was evident that males scored higher in the OA test and lower in the Err test compared to females.

**Table 9:** Comparison of the two gender groups (Mean Rank).

Sub-test	Male	Female	*p*
SA	52.61	48.63	0.494
OA	58.19	43.68	0.013
FA	46.07	54.42	0.151
Err	43.91	56.34	0.032

### 3.8 Impact of demographic variables

Multiple linear regression analysis suggested that when demographic variables age, education level, and gender were introduced into the linear regression model of SA and Err, SA was affected by years of education level and age, while Err was influenced by age and gender (Table 10).

**Table 10:** Impact of demographic variables.

Scale		B	S. E	*t*	*p*
Err	Age	0.281	0.036	7.728	0.000
	Gender	5.855	1.594	3.673	0.000
	Education level	−0.489	0.263	−1.863	0.066
	Constant	−6.977	3.300	−2.114	0.037
SA	Age	0.803	0.117	6.844	0.000
	Gender	1.753	4.053	0.432	0.666
	Education level	−2.088	0.668	−3.125	0.002
	Constant	60.167	13.194	4.560	0.000

### 3.9 Relevance to other attention tests

SA was negatively correlated with the net score of D-CAT and positively correlated with the error rate of D-CAT. It was also negatively correlated with DST forward and backward scores and SDMT scores. Err showed a positive correlation with the net score of D-CAT and a negative correlation with the error rate of D-CAT, DST forward and backward scores, and SDMT scores. OA and FA did not show significant correlation with other attention tests (Table 11).

**Table 11:** Relevance to other attention tests.

	SA		OA		FA		Err	
	*r*	*p*	*r*	*p*	*r*	*p*	*r*	*p*
D-CAT net score	−0.405	0.000	0.046	0.648	−0.045	0.658	−0.439	0.000
D-CAT error rate %	0.401	0.000	−0.048	0.635	−0.044	0.660	0.437	0.000
DST forward score	−0.624	0.000	0.035	0.732	−0.170	0.091	−0.458	0.000
DST backward score	−0.643	0.000	−0.046	0.646	−0.171	0.089	−0.417	0.000
SDMT score	−0.802	0.000	−0.059	0.557	−0.155	0.124	−0.529	0.000

### 3.10 Reliability testing

3.10.1 Re-testability of the BReViS test: Results showed that the correlation coefficients for SA, OA, and Err were all greater than 0.600, *p* < 0.001. Only the correlation coefficient for FA was below 0.6, *p* > 0.05, which was not statistically significant (Table 12).

**Table 12:** Re-testability of the BReViS test.

Index	Mean performance time (and SD) for the first test	Mean performance time (and SD) for the second test	*r*	*p*
SA	54.73 (15.10)	53.79 (14.27)	0.782	0.000
OA	29.60 (19.50)	22.17 (14.53)	0.659	0.000
FA	−1.57 (12.33)	0.67 (9.67)	0.110	0.564
Err	9.87 (6.17)	10.10 (5.55)	0.759	0.000

3.10.2 Internal Consistency Reliability: Cronbach’s alpha coefficient was 0.874, indicating high internal consistency reliability for the BReViS test.

### 3.11 Validity testing

3.11.1 Construct Validity: The Kaiser-Meyer-Olkin (KMO) measure and Bartlett’s test of sphericity results were 0.763 and 252.601 (P<0.001), respectively, indicating the scale was not very suitable for factor analysis.

3.11.2 Criterion Validity: In this study, the D-CAT was used as a criterion, and Spearman’s correlation analysis was used to calculate the correlation between BReViS’s SA and the net scores and error rates of D-CAT to evaluate the degree of criterion-related validity. The results showed that SA was significantly negatively correlated with the net score of D-CAT (*r* = −0.405, *p* < 0.001) and significantly positively correlated with the error rate of D-CAT (*r* = 0.401, *p* < 0.001), indicating the questionnaire has good criterion-related validity, as seen in Table 11.

### 3.12 Correlation between sub-tests

The correlation analysis of the results among the various sub-tests of the BReViS test indicated that the correlation coefficient between SA and Err was 0.532, and between OA and Err was-0.229, with *p* < 0.05, suggesting a certain degree of consistency between them, which contributes to ensuring the reliability of the scale. Meanwhile, the correlation between SA, OA, and FA was not high, indicating that the scale has excellent information content and structural validity, as seen in Table 13.

**Table 13:** Correlation between sub-tests.

Index	*r*	*p*
SA and OA	−0.004	0.971
SA and FA	0.074	0.462
SA and Err	0.532	0.000
OA and FA	−0.050	0.621
OA and Err	−0.229	0.022
FA and Err	−0.012	0.904

## 4 Discussion

Attention is a fundamental psychological concept, deeply embedded in cognitive processing, defined by the deliberate focusing on particular stimuli (van Es et al., 2018). This focusing elevates the level of awareness about these stimuli, epitomizing attention’s selective nature. Solso, MacLin M.K., and MacLin O.H. (2005) highlight that “the essence of attention lies in the concentration and focus of consciousness,” underlining attention’s critical role in selecting an item from an array of simultaneous stimuli or thought sequences (Baddeley, 1988). Selective attention, therefore, is the capacity to direct an individual’s finite processing resources toward a particular environmental aspect. This complex concept encompasses a range of processes, including spatial attention with its directional and focal elements (Carrasco, 2011). Such capability allows for the filtration of extensive information from the surroundings, facilitating the efficient usage of scarce cognitive resources.

Historically, attention has been a central theme in psychological studies, resulting in a plethora of theoretical frameworks and experimental methodologies. One of the most significant paradigms for investigating selective visual attention’s traits is visual search (Bacon and Egeth, 1997; Verghese, 2001; Wolfe, 2003). Everyday life is replete with visual search scenarios, whether it’s choosing products on supermarket shelves, animals searching for food amidst leaves, locating a friend in a large gathering, or playing visual search games (Wolfe, 2020). Clinical neuropsychology frequently employs visual search tasks (VST) to evaluate selective visual attention deficits in patients with neurological conditions (Senger et al., 2017). Standard VST protocols involve participants identifying a target among numerous stimuli, like figures or letters, assessing performance based on response accuracy and time (Wolfe et al., 2002).

Studies suggest that visual task outcomes are not just influenced by attention toward the target’s location (the spatial component) but also by adjusting the attention window according to the task requirements (the focal component) (Albonico et al., 2016), with each component operating independently (Castiello and Umilta, 1990; Carrasco and Yeshurun, 2009). Traditional VSTs, however, tend to neglect the influence of distractor arrangement and density on performance, thus failing to adequately capture the nuances of spatial attention (Weintraub and Mesulam, 1988; Mesulam, 2000). The BReViS assessment offers a refreshing alternative to conventional paper-and-pencil visual search tests by modifying the stimulus arrangement within the visual field, allowing for a comprehensive evaluation of selective visual attention and its distinct facets. Though previously utilized within the Italian demographic without undergoing thorough reliability and validity verification, this study introduces the BReViS test to the Mainland Chinese audience, undertaking a comprehensive examination of its reliability and validity among individuals aged 19 to 84.

### 4.1 Reliability testing

When a test has good reliability, it will yield almost the same scores for the same group of people at different times. The quality of reliability is also a prerequisite for validity testing. In this study, the test–retest reliability of the BReViS showed high correlation coefficients for three of the four sub-tests—SA, OA, and Err—on reassessment after two weeks. The test–retest results indicate that the BReViS test has good retest reliability, suggesting good temporal stability. The lack of statistical significance for FA in the correlation analysis may be due to the longer duration of this test, which may lead to fatigue in older participants resulting in unstable scores. Additionally, a higher Cronbach’s alpha coefficient indicates stronger internal consistency of the scale. It is generally considered that a Cronbach’s alpha coefficient greater than 0.7 indicates good consistency among items (Tavakol and Dennick, 2011). The results of this study show a total Cronbach’s alpha coefficient of 0.874 for the BReViS test, indicating high internal consistency reliability. It’s interesting to note that the average score for FA increased from −1.57 in the first test to 0.67 in the second, indicating a higher sensitivity to crowding in the latter. Research has shown that sensitivity to visual crowding is influenced by various factors that can affect an individual’s ability to distinguish objects in cluttered environments. These factors include contrast, eccentricity, visual acuity and age, spatial frequency, attention and perceptual learning, as well as stimulus similarity (Coates et al., 2013; Veríssimo et al., 2022). Therefore, factors such as the brightness of the room, the depth of color of the test figures, the position of the test paper in the field of vision, whether the participant is focused, has undergone perceptual learning, and the objects surrounding the test paper can all affect sensitivity to crowding. The variability in the results of the two tests in this study reminds us that these influences need to be more tightly controlled in future studies.

### 4.2 Validity testing

The Kaiser-Meyer-Olkin (KMO) measure and Bartlett’s test suggested that the structure of the BReViS test might not be well suited for factor analyses, but that there was some correlation between the BReViS measures. The correlation analysis among the results of each subt-est of the BReViS showed a correlation coefficient of 0.532 between SA and Err, and − 0.229 between OA and Err, with *p* < 0.05, indicating a certain level of consistency between them, which contributes to ensuring the reliability of the scale. However, the correlations among SA, OA, and FA were not high, suggesting that the scale has excellent information content and structural validity. Given that BReViS was developed to assess SA, this study employed the D-CAT as a criterion measure and found a significant correlation between SA and the D-CAT results, indicating good criterion-related validity.

### 4.3 The influence of age on BReViS

This study showed that age was significantly positively correlated with the sub-tests SA and Err. Multiple linear regression analysis suggested that SA is greatly influenced by age and education level, while Err is more influenced by age and gender. Therefore, age is a major factor influencing BReViS test results, which is consistent with the findings of the scale developers in the Italian population and previous research. The rank-sum test analysis across different age groups reveals that young adults significantly outperform both middle-aged and senior groups in selective attention tasks, making fewer errors. Additionally, the young-adult group demonstrate superior selective attention capabilities compared to those in the senior group. This pattern supports the notion that selective attention abilities undergo a pronounced growth during adolescence, which is then followed by a discernible decline as individuals age (Moore and Zirnsak, 2017). Neurophysiological alterations, observable through changes in the amplitude and latency of event-related potential (ERP) components, accompany this evolution in attention processing (Madden et al., 2007). Complementing these findings, functional MRI studies have identified a diminished activation in critical regions associated with visual attention control - namely, the bilateral fusiform gyrus, the right lingual gyrus, and the right precuneus-in elderly individuals when compared to their younger counterparts (Lyketsos et al., 1999; Lee et al., 2003).

### 4.4 The influence of education level on BReViS

This study found that years of education were negatively correlated with both SA and Err, and significant differences in SA and Err scores were also observed across different education level groups. Analysis using rank-sum tests across different educational attainment groups indicates that individuals with tertiary education (the college/university group and above) perform significantly better in selective attention tasks than those from the elementary (Mueller et al., 2008; Yehezkel et al., 2015), middle School and high school/vocational groups. They made fewer errors, suggesting a correlation between higher education levels and improved selective attention abilities. Studies have shown that individuals with higher levels of education often perform better on various cognitive tests (Lindenberger and Baltes, 1997; Hultsch et al., 1999), likely due to the enhanced cognitive strategies, problem-solving skills, and knowledge base provided by formal education. Additionally, higher education may mitigate the impact of aging on cognitive performance (Lee et al., 2003; Jones et al., 2006; Tun and Lachman, 2008; Marioni et al., 2012). Research by Stern et al. (2005) and others indicates that higher educational attainment can moderate the decline in reaction and attention abilities due to aging and lower the risk of dementia (Bell et al., 2006), partly because cognitive reserve accumulation improves brain network efficiency (Rubia et al., 2010). These findings highlight the importance of considering educational background when interpreting cognitive assessment results.

### 4.5 The influence of gender on BReViS

In this study, the SA index was influenced by age and educational level, but no significant gender differences were observed. Gender was positively correlated with the Err index and negatively correlated with the OA index, with significant differences between genders, indicating that females committed more total errors than males. Males had higher OA scores than females, suggesting that males in the visual search process rely on exogenous cues to perform tasks correctly and are less likely to follow effective endogenous strategies. This is consistent with the observations made by the authors in a normal Italian population. The differences in OA scores between males and females may be related to the activation of different brain regions during the execution of spatial selective attention tasks. Males show increased activation in the left hemisphere’s inferior parietal lobule, while females show significant activation in the right hemisphere’s inferior frontal gyrus, insula, caudate, and temporal areas (de Fockert et al., 2001; Boi et al., 2011), which may be related to the modulation by estrogen and testosterone (Oberauer, 2019). Additionally, FA was not observed to be affected by gender, age and years of education in this study, which is in line with the results of the most recent application of the scale, i.e., crowding did not worsen with age (Pegoraro et al., 2024), and these findings are consistent with previous studies (Malavita et al., 2017; Shamsi et al., 2022).

### 4.6 The correlation between BReViS and other attention scales

SA was significantly positively correlated with the cancellation time and error rate in the D-CAT and significantly negatively correlated with the net score of cancellation. Err was negatively correlated with the net score of cancellation and positively correlated with the cancellation error rate. These results indicate that BReViS’s SA and Err have good consistency with the D-CAT in assessing selective attention in the normal population.

Research demonstrates that enhancing selective attention significantly improves test outcomes in immediate memory capabilities (Plebanek and Sloutsky, 2019). For instance, within the context of the DST, superior selective attention enables individuals to recall and reproduce digit sequences with greater accuracy, thus exhibiting an increased memory capacity. This study reveals a negative correlation between SA and Err with the scores of forward and backward span in the DST, offering a crucial insight: higher scores of SA and Err indicate weaker selective attention, an increased error rate, and a noticeable decline in the subjects’ immediate memory capacity. This finding highlights the close interrelation among immediate memory, selective attention, and cognitive efficiency, suggesting that individuals with a larger immediate memory capacity can more effectively resist distractions, thereby reducing error rates (Posner and Petersen, 1990; Rayner, 1998; Ku, 2018). In clinical practice, this correlation is important to identify and assess deficits in attention, working memory, or other cognitive functions.

The negative correlation between SA and Err with scores on the SDMT unveils a significant cognitive phenomenon: there is a direct correlation between elevated selective attention and increased efficiency of visual scanning, speed of information processing, and hand-eye coordination. Selective attention, a critical dimension of attention management, involves filtering task-relevant information from the environment while disregarding irrelevant distractions (De la Torre et al., 2015). The efficacy of selective attention depends to a large extent on the efficiency of visual scanning, a crucial aspect because it requires the individual to quickly localize and identify key targets among numerous visual stimuli (Reigal et al., 2019). Furthermore, the acceleration of information processing speed is a key factor in enhancing the efficiency of selective attention, allowing individuals to recognize important information within shorter durations and respond accordingly (Posner, 1980). In tasks requiring rapid identification of visual information followed by corresponding physical actions, exceptional hand-eye coordination markedly improves the precision and efficiency of task execution (Castiello and Umilta, 1990). Thus, the effective concentration of selective attention on specific stimuli or tasks is supported by an individual’s performance in terms of a combination of speed of information processing, visual scanning ability, and hand-eye coordination. The improvement of these cognitive abilities not only further enhances the performance of selective attention but also, reciprocally, enhances the operational efficacy of these cognitive functions, thereby creating a positive feedback loop. This phenomenon offers profound insights into how individuals process information efficiently in complex environments within the domain of cognitive science.

The allocation of attentional resources in space involves two distinct processes: the orienting process, which selectively concentrates on specific aspects of the environment while ignoring others. The OA index reflects orienting ability, influenced by factors like stimulus salience, personal interests or goals, and the presence of attention-directing cues (Chun et al., 2011). The focusing process narrows attention to a specific area or object, acting like a magnifying glass, allowing selective concentration on a limited spatial area (Turatto et al., 2000; Chun et al., 2011). The FA index reflects focusing ability. Some studies suggest that focusing and orienting may vary based on visual conditions (Turatto et al., 2000). This research found no significant correlation between OA and FA with DST and SDMT, suggesting that orienting and focusing abilities might not be affected by immediate memory capacity, information processing speed, visual scanning ability, and hand-eye coordination skills.

## 5 Conclusion

The BreViS test, demonstrating good reliability and validity, is adept for application across a broad age range (19 to 84 years) within the general population, assessing not only selective attention but also gauging capacities in immediate memory, information processing speed, visual scanning, and hand-eye coordination. The influence of demographic variables such as age, gender, and education level on test outcomes underscores the necessity for nuanced interpretation of results in research and clinical settings.

## Data availability statement

The original contributions presented in the study are included in the article/supplementary material, further inquiries can be directed to the corresponding author.

## Ethics statement

The studies involving humans were approved by the Ethics Committee of the Third Affiliated Hospital of Soochow University. The studies were conducted in accordance with the local legislation and institutional requirements. The participants provided their written informed consent to participate in this study.

## Author contributions

YS: Writing – original draft. YZ: Writing – review & editing.
